# Tocilizumab-coated solid lipid nanoparticles loaded with cannabidiol as a novel drug delivery strategy for treating COVID-19: A review

**DOI:** 10.3389/fimmu.2023.1147991

**Published:** 2023-03-22

**Authors:** Aleksandra Zielińska, Piotr Eder, Jacek Karczewski, Marlena Szalata, Szymon Hryhorowicz, Karolina Wielgus, Milena Szalata, Agnieszka Dobrowolska, Atanas G. Atanasov, Ryszard Słomski, Eliana B. Souto

**Affiliations:** ^1^ Institute of Human Genetics, Polish Academy of Sciences Poznan, Poznan, Poland; ^2^ Department of Gastroenterology, Dietetics, and Internal Diseases, Poznan University of Medical Sciences, Poznan, Poland; ^3^ Department of Environmental Medicine/Department of Gastroenterology, Human Nutrition and Internal Medicine, Poznan University of Medical Sciences, Poznan, Poland; ^4^ Department of Biochemistry and Biotechnology, Poznań University of Life Sciences, Poznań, Poland; ^5^ Department of Pediatric Gastroenterology and Metabolic Diseases, Poznan University of Medical Sciences, Poznan, Poland; ^6^ Department of Biotechnology, Institute of Natural Fibres and Medicinal Plants National Research Institute, Poznan, Poland; ^7^ Institute of Genetics and Animal Biotechnology, Magdalenka, Poland; ^8^ Institute of Neurobiology, Bulgarian Academy of Sciences, Sofia, Bulgaria; ^9^ Department of Pharmacognosy, University of Vienna, Vienna, Austria; ^10^ UCIBIO – Applied Molecular Biosciences Unit, MEDTECH, Laboratory of Pharmaceutical Technology, Department of Drug Sciences, Faculty of Pharmacy, University of Porto, Porto, Portugal; ^11^ Associate Laboratory i4HB - Institute for Health and Bioeconomy, Faculty of Pharmacy, University of Porto, Porto, Portugal

**Keywords:** COVID-19, solid lipid nanoparticles (SLN), tocilizumab (TCZ), cannabidiol (CBD), cytokine storm, oral drug therapy

## Abstract

Commonly used clinical strategies against coronavirus disease 19 (COVID-19), including the potential role of monoclonal antibodies for site-specific targeted drug delivery, are discussed here. Solid lipid nanoparticles (SLN) tailored with tocilizumab (TCZ) and loading cannabidiol (CBD) are proposed for the treatment of COVID-19 by oral route. TCZ, as a humanized IgG1 monoclonal antibody and an interleukin-6 (IL-6) receptor agonist, can attenuate cytokine storm in patients infected with SARS-CoV-2. CBD (an anti-inflammatory cannabinoid and TCZ agonist) alleviates anxiety, schizophrenia, and depression. CBD, obtained from Cannabis sativa L., is known to modulate gene expression and inflammation and also shows anti-cancer and anti-inflammatory properties. It has also been recognized to modulate angiotensin-converting enzyme II (ACE2) expression in SARS-CoV-2 target tissues. It has already been proven that immunosuppressive drugs targeting the IL-6 receptor may ameliorate lethal inflammatory responses in COVID-19 patients. TCZ, as an immunosuppressive drug, is mainly used to treat rheumatoid arthritis, although several attempts have been made to use it in the active hyperinflammatory phase of COVID-19, with promising outcomes. TCZ is currently administered intravenously. It this review, we discuss the potential advances on the use of SLN for oral administration of TCZ-tailored CBD-loaded SLN, as an innovative platform for managing SARS-CoV-2 and related infections.

## 1 Highlights

- Tocilizumab (TCZ) attenuates cytokine storm in SARS-CoV-2-infected patients;
- Cannabidiol (CBD) promotes alleviation of anxiety, schizophrenia and depression;
- High levels of CBD from *Cannabis sativa* L. may be used to modulate angiotensin-converting enzyme II (ACE2) expression in SARS-CoV-2 target tissues;
- Dual TCZ and CBD-loading in lipid nanoparticles may ameliorate lethal inflammatory responses in COVID-19 patients;
- Lipid nanoparticles are suitable for orally-administered TNF-α inhibitors.

## 2 COVID-19 – Current therapies and promising drugs

Coronavirus disease-2019 (COVID-19) is caused by the severe acute respiratory syndrome (SARS) coronavirus-2 (SARS-CoV-2). It was first reported in Wuhan, China, in November 2019, when the outbreak was dated. Based on scientific reports, this acute infection is related to a cytokine storm, causing symptoms such as fever, cough, and muscle pain. In most severe cases, bilateral interstitial pneumonia with ground-glass opacity and focal chest infiltrates can be observed by using computerized tomography scans (1).

Despite the urgent need for specified therapeutic intervention, there are no effective antiviral drugs or vaccines against SARS-CoV-2. In October 2020, FDA approved remdesivir as the first promising antiviral drug to treat COVID-19 patients (2, 3). Previously, this antiviral drug has been applied to treat hepatitis C and was also used against Ebola. In the EU, remdesivir is now licensed to treat COVID-19 in adults and adolescents with pneumonia requiring supplemental oxygen (4).

Studies demonstrate that hospitalized COVID-19 patients with a lower respiratory tract infection in the remdesivir group recovered faster than patients in the placebo group (5). However, the clinical status of the patients within the 10-day course of remdesivir did not have any statistical improvement compared to standard care at 11 days after initiation of treatment in the case of moderate COVID-19. On the other hand, patients randomized to a 5-day treatment with remdesivir have shown a statistically significant difference compared to standard care. The obtained clinical importance was unreliable (6). To sum up, the first randomized trial indicated that remdesivir has no significant clinical values. In contrast, the numerical reduction in time to clinical improvement points out the need for more research investment (7).

Even though hydroxychloroquine, lopinavir/ritonavir, and interferon were also proposed against SARS-CoV2 (8), research is still ongoing on more effective treatments. Among available therapeutic regimens, the most common drugs are those used for autoimmune diseases, antiviral agents, and antibodies from people who have recovered from COVID-19. It is worth underlining that due to the reproduction of viruses, an efficient antiviral drug should be able to target the specific part of its life cycle necessary. Moreover, antiviral agents must be able to kill viruses without killing human cells.

Still, plenty of ongoing clinical trials of COVID-19 treatment are performed worldwide. In June 2020, the European Medicines Agency (EMA) announced negotiations with the developers of 132 potential COVID-19 therapies (9). Different drugs listed in **Table 1** have been mentioned among possible medications for treating COVID-19.

**Table 1:** Identification of the potential drugs for treating COVID-19.

	Drug Name	Previous use	Evidence of efficacy against COVID-19	Ref.
ANTIVIRALS	Remdesivir	Hepatitis C and Ebola	Remdesivir was higher-efficient than placebo regarding the time shortened to recovery in hospitalized COVID-19 patients.	(4, 5, 8)
	Chloroquine/hydroxychloroquine	Malaria	Daily hydroxychloroquine is ineffective in protecting exposed hospital-based healthcare workers from contracting SARS-CoV-2 infection, but the trial has been suspended.	(10, 11)
	Lopinavir/ritonavir combination	HIV infection, when combined with other antiretrovirals	Initial results may suggest that the impact of lopinavir/ritonavir has inconsiderable or no effect on mortality in hospitalized COVID-19 patients.	(12, 13)
	Favipiravir	Influenza, coronavirus (*in vitro*)	Favipiravir combined with tocilizumab can effectively reduce the mortality of COVID-19.	(14, 15)
	Umifenovir	Influenza (Russia and China)	There is no supporting evidence of use in patients with COVID‐19; no evidence of improvement. SARS-CoV-2 clearance in non-ICU patients. Need for randomized control clinical trial for efficacy assessment of umifenovir.	(16, 17)
	Ribavirin	Hepatitis C, respiratory syncytial virus (RSV), and bronchiolitis	Early treatment with triple antiviral therapy, consisting of IFN beta-1b, lopinavir-ritonavir, and ribavirin, reduces the duration of viral shedding in patients with mild to moderate COVID-19. Only ribavirin did not lower the mortality rate compared with the control group.	(18–20)
	Molnupiravir (also known as MK-4482 or EIDD-2801)	RNA viruses (broad spectrum), including Influenza, coronaviruses (SARS, MERS, and SARS-CoV-2)	Trials have shown that it may reduce mortality and speed recovery in COVID-19 patients.	(21)
	Niclosamide	Antihelminthic drug; effective agent against various viral infections, such as SARS-CoV, MERS-CoV, ZIKV, HCV	Niclosamide has been shown to have inhibitory activity on the replication of SARS-CoV. It enables the entry of SARS-CoV-2 by altering endosomal pH and restraining virus replication by inhibiting autophagy.	(22–24)
	Oseltamivir	Influenza A and B	Early oseltamivir administration, combined with antibacterial therapy, may lower the duration of fever in COVID-19-suspected outpatients without hypoxia.	(25)
IMMUNE MODULATORS	Dexamethasone	Reduction of inflammation by mimicking anti-inflammatory hormones produced by the body	Dexamethasone reduces 28-day mortality among those receiving invasive mechanical ventilation or oxygen at randomization. However, it is not among patients not receiving respiratory support.	(26)
	Hydrocortisone	Reduction of inflammation by mimicking anti-inflammatory for adrenocortical insufficiency, rheumatoid arthritis, dermatitis, asthma, chronic obstructive pulmonary disorder	Evidence regarding corticosteroid use against SARS-CoV-2 is limited; low-dose hydrocortisone has not significantly prevented death or continued respiratory support for severe COVID-19 patients.	(27, 28)
	Azithromycin	Macrolide antibiotic; activity against, e.g., influenza A and zika	In patients with severe COVID-19, azithromycin, including caring treatment with hydroxychloroquine, has not improved clinical outcomes.	(29)
	Tocilizumab	Rheumatoid arthritis, Systemic juvenile idiopathic arthritis	Tocilizumab reduces the need for non-invasive ventilation, improving the clinical diagnosis of COVID-19 patients and reducing the risk of death by day 14 (although not mortality by day 28).	(14, 30)
	Sarilumab	Rheumatoid arthritis	Sarilumab has shown unclear efficacy results in the ongoing trial of hospitalized patients with severe or critical respiratory illness secondary to COVID-19.	(31, 32)
	Canakinumab	Inhibits IL-1; recommended to treat periodic fever syndromes and gouty arthritis	It has been used to treat cytokine release syndrome in severely ill COVID-19 patients.	(33, 34)
	Anakinra	Inhibits IL-1; for the treatment of rheumatoid arthritis	Using to reduce the cause of acute respiratory distress syndrome (ARDS) in COVID-19 patients.	(35, 36)
	Baricitinib	Janus-associated tyrosine kinase (JAK1 and JAK2 inhibitor); Rheumatoid arthritis	Baricitinib can reduce the cytokine-release syndrome associated with COVID-19; It has reduced the COVID-19 mortality rate in a retrospective multicenter trial.	(37, 38)
	Ruxolitinib	Inhibitor of JAK 1/JAK 2 and indicated for specialist treatments, such as in blood diseases	Using ruxolitinib, a direct block of the SARS-CoV-2 enters the cell has been noticed. Although there is a risk of adverse effects (opportunistic infections to the immunosuppression must also be considered), it significantly impacts overcoming complications due to immune hyperactivation by the JAK/STAT signaling pathway.	(39–41)
	Acalabrutinib	Lymphocytic leukemia	It decreases inflammation and improves outcomes in severe COVID-19 patients.	(42)
	**Ravulizumab**	**Regularly used in blood diseases where complement activation destroys red blood cells.**	**III-phase of ongoing randomized and controlled trials to assess the safety and efficacy of ravulizumab in COVID‐19 patients with severe pneumonia or ARDS.**	(43, 44)
	**Infliximab**	**Rheumatoid arthritis, Inflammatory bowel disease (IBD)**	**Under investigation on its use in the management of inflammation associated with COVID-19.**	(45)
	**Adalimumab**	**Rheumatoid arthritis, Inflammatory bowel disease (IBD)**	**Recent studies have shown that COVID-19 patients were more rarely treated in hospitals due to taking anti-TNF drugs for other conditions.**	(46, 47)
	**Namilumab**	**Rheumatoid arthritis; Ankylosing spondylitis**	**It has been shown to have already approved the safety profile from its use in ongoing clinical trials.**	(47–49)
	**Otilimab**	**Arthritis**	**IV-phase of ongoing randomized, double-blind, placebo-controlled trials to determine the safety and efficacy of otilimab in COVID‐19 patients with severe pneumonia.** **It has already shown promising results during the initial developmental phases.**	(48, 50)
	**Lenzilumab**	**Recombinant monoclonal antibody targeting human GM-CSF, with a potential role in the pathogenesis of COVID-19–related immune hyper-response**	**III-phase of therapy randomized and controlled trials to determine its use as sequenced therapy with CAR-T treatments. Obtained results have shown the higher effectiveness of tocilizumab than lenzilumab in managing this cytokine-mediated syndrome in treating COVID-19.**	(48, 51)
	**Leronlimab**	**A promising therapy in the treatment of triple-negative breast cancer and HIV infection**	**Ongoing trials to detect the safety and efficacy of leronlimab in COVID‐19 patients.**	(52)
	**Bamlanivimab (LY-CoV555)**	**A potent neutralizing IgG_1_ monoclonal antibody to target the spike protein of SARS-CoV-2**	**One of three doses (2800 mg) of bamlanivimab can accelerate the natural decline in viral load over time in mild or moderate COVID-19 patients.**	(53–55)
	**Etesevimab** **(LY-CoV016)**	**Targeting SARS-CoV-2 spike protein and blocking the binding of the virus to the ACE2 host cell surface receptor**	**Ongoing trials to identify the safety and efficacy of etesevimab in patients with mild to moderate COVID-19. *In vivo* studies have shown that it may be efficient for prophylactic and therapeutic venues against SARS-CoV-2 infection by reducing viral load, symptoms, and COVID-19-related hospitalization.**	(54)

Bold values refer to ongoing clinical trials.

The need for drug repurposing has increased to prompt an efficient way to fight against SARS-CoV-2. Various drug repurposing screenings have chosen several potential drug candidates against COVID-19, but no one is fully efficient.

In this review, we discuss the studies that have implemented tocilizumab (TCZ) as an anti-IL-6 receptor antibody in COVID-19 treatment, proposing a new TCZ-coated platform for the targeted delivery of CBD. Many already published results indicate that the combination of dual delivery TCZ and CBD may aid in the recovery of patients with COVID-19 and reduce mortality. A novel approach is therefore discussed here, exploiting opportunities associated with linking nanocarriers loaded with TCZ and its agonist – CBD. Both drugs can inhibit IL-6, a major inflammatory cytokine involved in cytokine release syndrome (CRS) in various inflammatory conditions (56). Therefore, this dual-drug delivery system may have a crucial meaning in the mechanism of SARS-CoV-2 infections.

## 3 Clinical view of COVID-19

**Figure 1:**
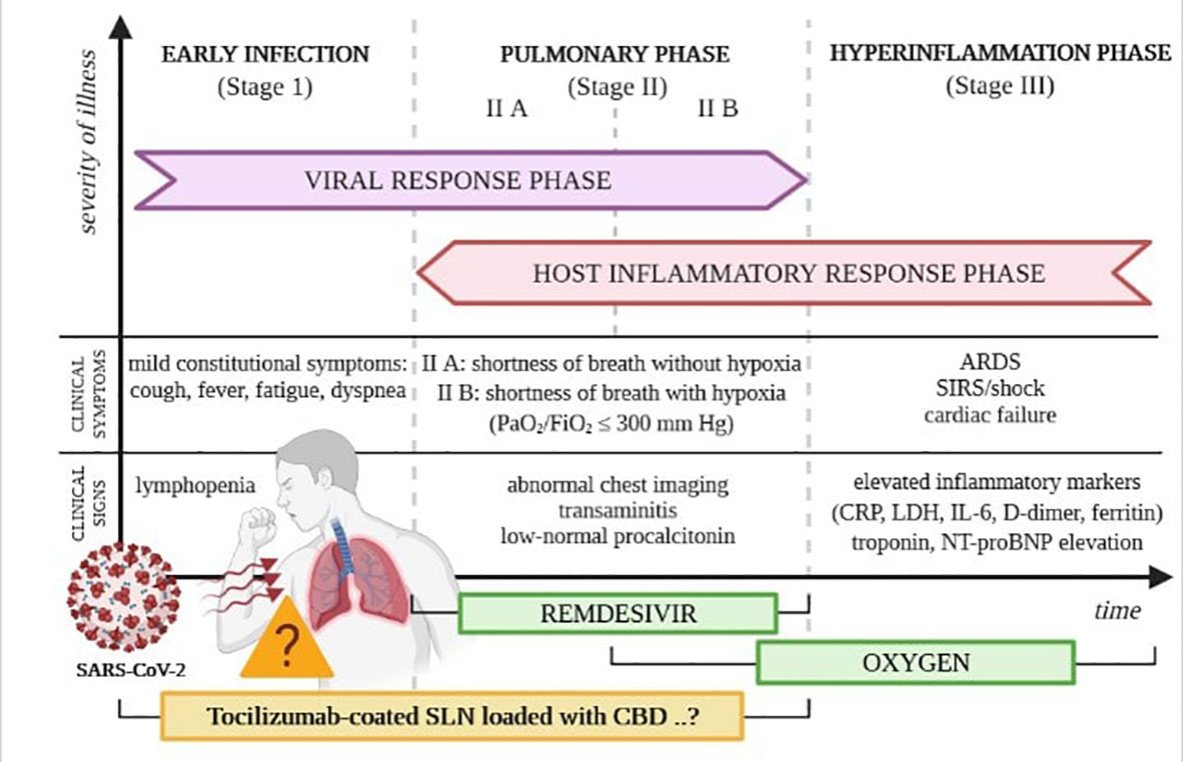
Phases of the clinical course of COVID-19. Own drawing, based on (60).

The SARS-CoV-2 virus has already infected millions of people worldwide and has led to numerous deaths. COVID-19 was firstly recognized and described in November 2019 in Wuhan (Hubei Province, China) during a series of cases that initiated the pandemic of this disease (57). The transmission of infection is primarily by droplet infection (58). Still, the SARS-CoV-2 virus is also noted in the stool, suggesting that the gastrointestinal tract is the second - after the respiratory system - target site for initial viral replication (59). The natural course of COVID-19 can be split into three stages of development: early, pulmonary, and hyperinflammatory. Each phase, presented in **Figure 1**, has slightly different clinical characteristics and minor differences in the proposed treatment algorithms (60). Many infected patients are asymptomatic, augmenting the spread of the virus (61). When symptomatic, cough, fever, fatigue, and dyspnoea are the most frequent manifestations of COVID-19; however, some people develop serious complications resulting in death. People with reduced immunity are at the highest risk of more severe side effects attributed to the infection, such as dysfunction of specific organs or even respiratory failure (61).

In many cases, COVID-19 can cause some other less typical clinical manifestations (57). Very often, anxiety is the accompanying symptom. As the expression of specific receptor proteins for the SARS-CoV-2 virus is exceptionally high in intestinal epithelial cells, symptoms at the gastrointestinal level (e.g., diarrhea, abdominal pain, and nausea) are also commonly reported, especially in the early stages of the disease (59, 62). The risk factors for a severe course of the illness with dynamic progression to the hyperinflammatory phase, the clinical manifestation of which is acute respiratory distress syndrome, circulatory failure, and shock, are still not fully understood. The most crucial pathophysiological phenomenon responsible for these processes is the cytokine-associated toxicity resulting from SARS-CoV-2 virus infection (58). One of the critical directions of research into an effective COVID-19 therapy is the search for drugs that can inhibit and prevent the uncontrolled production of pro-inflammatory cytokines, showing significant potential to damage tissues, including respiratory diseases. Cytokine-associated toxicity or cytokine release syndrome (CRS) is mainly associated with pro-inflammatory cytokine IL-6 released in severe COVID-19 infections. Cytokine IL-6 initiates the CRS in the MAPK/NF-κB-IL-6 or JAK-STAT pathway (63), and as an infection trigger, IL-6 has been associated with the symptom progression in this disease (64). The cytokine-associated toxicity has been regarded as the most typical marker of the severity of COVID-19 infection and high mortality risk (65).

As the number of patients infected with the SARS-CoV-2 virus continues to grow worldwide, there is still a need to introduce an effective therapy that can provide effective treatment in all phases of the infection and especially prevent the progression of COVID-19 into clinical forms that constitute a direct threat to the patient’s life.

## 4 Gene delivery and therapy in SARS-CoV-2 infection

**Figure 2:**
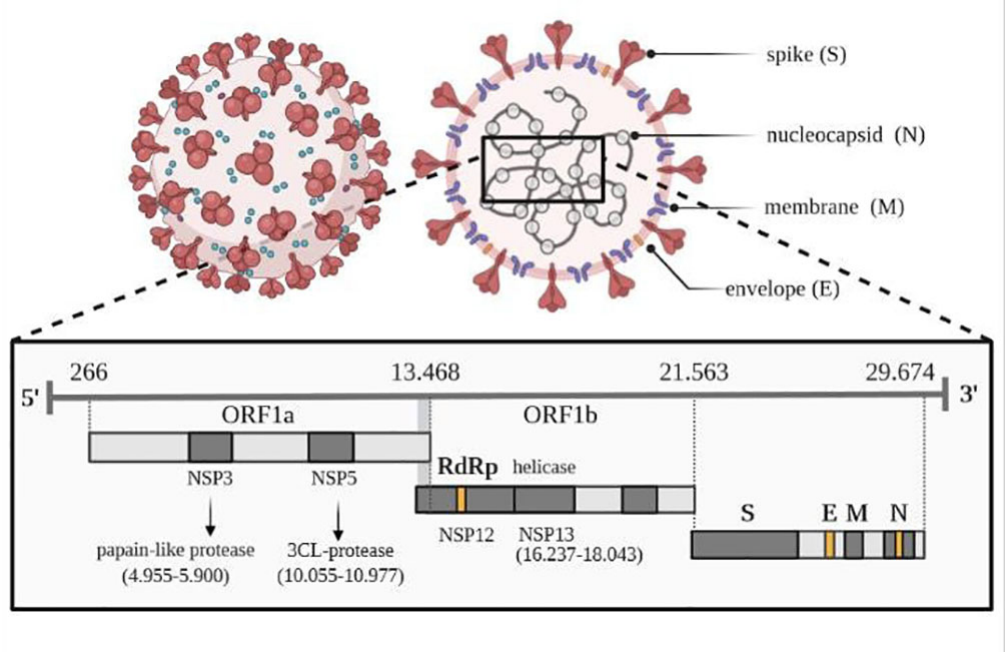
Viral genome of SARS-CoV-2. Own drawing, based on (66).

The genome sequence of SARS-CoV-2, shown in **Figure 2**, shows a very high similarity to SARS-CoV-2. Therefore, 3D homology sequence lines were used to analyze potential antiviral properties based on databases of over 32 000. Recognized medicinal plants and substances used in Chinese medicine are listed in **Table 2** (67).

**Table 2:** Selected plant substances with potential antiviral activity.

Common name	Latin name	Active compound
Edible amaranth Chinese amaranth Amarante Douteuse	*Amaranthus tricolor* L.	Amaranthin
Siebold Ash Japanese Flowering Ash Chinese Flowering Ash	*Fraxinus sieboldiana* Blume	Calceolarioside B
Indian gooseberry	*Phyllanthus emblica* L.	(2S)-Eriodictyol 7-O-(6″-O-galloyl)-beta-D-glucopyranoside
Common bean	*Phaseolus vulgaris* L.	3,5,7,3′,4′,5′-hexahydroxy flavanone-3-O-beta-D-glucopyranoside
Chinese liquorice Gan cao	*Glycyrrhiza uralensis* Fisch.	Licoleafol
Wax myrtle Southern wax myrtle, southern bayberry	*Myrica cerifera* L.	Myricitrin
Tea tree Tea Plant Assam tea	*Camellia sinensis* (L.) Kuntze	Myricetin 3-O-beta-D-glucopyranoside
Mojave indigo bush California Indigobush Mojave Dalea,	*Psorothamnus arborescens* (Torr. exA.Gray) Barneby	5,7,3′,4′-Tetrahydroxy-2’-(3,3-dimethylallyl) isoflavone
Marubio oscuro	*Hyptis atrorubens* Poit.	Methyl rosmarinate

The genome of the SARS-CoV-2 virus distinguishes genes encoding structural proteins that make up the virus particle, including spike proteins (S protein) essential for infection, as well as envelope proteins (E), nucleocapsid proteins (N) and membrane proteins (M) (66). The SARS-CoV-2 virus also contains sequences encoding non-structural proteins (NSP), which inhibit the host’s innate immune response to infection through the activity of papain-like protease (PLP) encoded by the NSP3 sequence and 3CL protease encoded by the NSP5 sequence, located in the gene designated as ORF1a. The virus has its RNA polymerase (RdRP) that is RNA-dependent (NSP12) and an RNA helicase (NSP13) located within the ORF1b gene. One of the tasks of the 16 non-structural proteins involves the transcription and translation of the genome (RTC replication-transcription complex).

Substances with an antiviral activity that inhibit the main proteases of the SARS-CoV-2 virus (Mpro) can be searched among bioactive compounds from medicinal plants using a molecular docking strategy. Khaerunnis et al. informed that nelfinavir and lopinavir can treat SARS-CoV-2 infection. In addition, the main protease activity is inhibited by apigenin-7-glucoside, curcumin, demethoxycurcumin, catechin, epicatechin-gallate, and oleuropein, luteolin-7-glucoside has been shown, which can be used in therapy (68).

Further work has identified other low-risk drugs that can inhibit the activity of the COVID-19 protease due to its ability to bind to it. They are also characterized by high affinity, suggesting the potential for use in treating viral infection. Among the proposed substances, bilobalide and citral can be distinguished, in addition, to forskolin, ginkgolide A, menthol, or noscapine, salvinorin A and beta selinene and thymoquinone (69). An interesting substance of plant origin is cannabidiol (CDB), which may be used for immunological health support and possible substance protection from infection. The anti-inflammatory effect was found in various immune-mediated disorders, including autoimmune conditions and neurodegeneration. Since cannabidiol can support the body during infection against pathogens, it is assumed that it can play a similar role in COVID-19 disease. Cannabidiol reduces the secretion of cytokines, leading to a decrease in the level of chemokines.

Moreover, the anti-inflammatory effect is associated with limiting cell-mediated immunity with the participation of effector T cells. Similarly, in the central nervous system, it also modulates the activity of microglial cells (70, 71). This information is interesting within the problem of cytokine storm, which strongly influences the odds ratio of survival of affected COVID-19 patients. The main conclusion of many papers is the need for proper analysis of the influence of cannabidiol and tetrahydrocannabinol (THC) as potential therapeutic substances with antiviral properties (71–73).

Patients infected with SARS-CoV2 are treated with various drugs that reduce excessive inflammation associated with the enormous secretion of cytokines. The most commonly used are interleukin inhibitors, Janus kinase inhibitors, corticosteroids, convalescent Plasma, interferons, nitric oxide, statins, and adjunctive nutritional therapies, including zinc and vitamin D (47, 74, 75).

One of the drugs under investigation is tocilizumab (Actemra). This IL-6 inhibitor participates in several phase III clinical trials, all randomized (for participants and investigators) and double-blind. For example, clinical trials in patients with COVID-19 pneumonia, COVACTA, and EMPACTA, included an analysis of tocilizumab and a placebo, and REMDACTA additionally included an analysis of remdesivir. Preliminary results of clinical trials are encouraging (76–78).

An essential part of any therapy is the successful delivery of a drug or new gene construct, for example, based on genome editing, to the destination. Still, the big challenge is how to deliver the therapeutic protein to the specific compartment of the target cell (79). Nanocarriers based on lipids, polymers, graphene, or gold have been proposed. It is assumed that using nanoparticles based on lipids and polymers does not stimulate the immune system’s response and does not cause additional problems for cells or tissues (80).

After administration *via* systemic injection or by oral route, numerous obstacles must be overcome before reaching specific cell types or cellular compartments. One possibility is to put a drug, nucleic acids, or proteins into a nanoparticle shell, preventing aggregation, immune clearance, kidney removal, or premature release. It is assumed that the carrier, e.g., non-viral nanoparticle, and the transferred payload/cargo should be compatible in electrostatic charge. On the surface of the shell, additional targeting ligands may improve delivery precision. The next step will cover crossing the cell membrane of the target cell, which may be overcome by bombardment, using guiding peptides, or even *via* endocytosis. The developed formulations have to escape endosomes. After that, they may stay in the cytoplasm or journey to the nucleus. At each point, the risk of degradation has to be considered. For the delivery of nucleic acids, viral vectors (e.g., lentiviruses and adeno-associated viruses), are used. Due to problems related to their limited loading capacity, the possibility to increase immune response connected with mutagenesis and carcinogenesis, and issues with up-scaling, other delivery systems are being proposed. Nonviral vectors use lipids and/or polymers as nanocarriers and may deliver large cargo (81, 82). The choice of a virus-based or a non-viral vector will depend on the load we want to transfer to the cell rather than on the vector itself.

For lipid-based nanoparticles, synthetic lipids containing disulfide bonds, which will break after exposition to the reducing environment in the cell and release the nanoparticle contents, have been proposed (81). Another study used a covalently cross-linked thin polymer carrier that can be removed by glutathione (GSH) to deliver a Cas9 ribonucleoprotein complex for *in vivo* genome editing. The presence on the surface of the additional nanocapsule peptides may guide the whole structure to the destination cells. Inside the cell, cytosolic glutathione may disrupt the shell releasing the cargo (82).

Biocompatible carriers such as lipid and polymeric nanoparticles, peptide/protein and messenger RNA complexes, and other biomaterials have recently been used for *in vivo* protection and delivery of various loads (83, 84). For the formulation of lipid-like nanoparticles like cationic lipids, such as DOTAP and DOTMA, and ionizable lipid derivatives such as TT3, 5A2-SC8, LP-01, cKK-E12, and A18-Iso5-2 DC18 to improve the efficiency and lowering toxicity *in vivo* (83).

Polymeric carriers are of great interest due to their ease of uptake by cells, the ability to combine with proteins, and the ability to form complex systems with biologically active biodegradable substances. Carriers often show the ability to bind to the cell surface; such endocytic uptake is due to their functionalization/modification. Perfluorocarbon nanoemulsions (PFCs) more and more often are used in medicine, enabling the response to emerging stimuli, taking part in the active prevention and control of hemorrhage, actively participating in the transport of oxygen as synthetic artificial blood or in tissue ischemia (79). Such substances are sensitive to ultrasound, which allows them to be tracked and activate selected proteins at the expected target sites. This strategy may find application in the tightly controlled delivery of antibodies.

Cell and gene therapies aim to replace a defective gene. New medicines can be used in the treatment of a whole range of common diseases of great social importance, often with a complex genetic background (e.g., cancer, heart disease, or diabetes), conditioned by single genes (e.g., cystic fibrosis, hemophilia) and infections caused by viruses (e.g., AIDS). Cell and gene therapies still need to be fully commercialized and may be available only as part of a clinical trial, but already some treatments are marketed. Gene therapy possibility as the replacement of a diseased gene variant was mentioned in 1972. Still, it has taken years since the first gene therapy, Gendicine, for skin cancer was commercialized in China in 2003. FDA approved the first gene therapy in 2017. Since the first gene therapy patient Jesse Gelsinger died in 1999 (the ornithine transcarbamylase gene using a recombinant adenovirus), gene therapy may become a reality due to knowledge of the human genome, availability of precise and efficient tools for genes edition, and using of better delivery methods. Currently, several gene therapy products are commercially available, approved by the relevant agencies (Europe: European Medicines Agency, U.S.: Food and Drug Administration); advanced therapy medicines include Kymriah, Luxturna, Tecartus, Yescarta, as well as Zolgensma. Gene therapy aims to cure diseases caused by defined single-gene mutations mainly. Cell-specific delivery and immunogenicity remain challenges in gene therapy. Gene or drug transfer can be based on two strategies that enable the introduction of permanent changes by incorporating them into the genome or obtaining a transient effect; the first often use modified viruses, and the second can use lipid nanoparticles. After using adenovirus as a delivery vector, nowadays mainly for *in vivo* gene therapy, adeno-associated viruses (AAVs) are used and *in vitro* modification lentiviruses, for example, in chimeric antigen receptor (CAR) T cells (85, 86). Scientists also focused on decreasing the toxicity of viral vectors by limiting viral dosage by using capsids with increased efficiency of cell penetration, or the possibility of modifying virus expression specific for target tissues, or increasing the purity of the virus injected.

Gene therapy is not only strictly focused on gene delivery; there are attempts to cure polygenic disorders by delivering proteins for disease treatment (87). Another approach involves using a modifier gene platform and providing a functional copy of the gene encoding the retina-specific nuclear receptor *NR2E3* using the Adeno-Associated Virus AAV platform for gene therapy instead of correcting the damaged gene (88), changing disease phenotype.

It is also interesting to use specific receptors activated only by dedicated active substances (DREADD) for non-invasive and longitudinal tracking of neuron activity (89). Gene therapy may establish resistance to infectious diseases, and fragments of the SARS-CoV-2 sequence may be delivered within an AAV capsid (vaccine AAVCOVID). Production of antibodies against the adeno-associated virus may be prevented by endopeptidase imlifidase (IdeS) expression without disrupting B lymphocytes (90). The availability of therapy is limited in price.

Gene therapies are available on the market offer insertion of correct genes: Gendicine (China), Glybera (EU), and Imlygic (China, US, and EU); delivery of the DNA for drug production: Holoclar (EU), Kymriah (US and EU), Luxturna (US and EU), Strimvelis (EU), Yescarta (US, EU, in China under clinical trials), Zolgensma (US), Zynteglo (EU); gene interference: Defitelio (US and EU), Exondys 51 (US), Kynamro (US), Macugen (US) and Spinraza (US).

Gene therapy strategies include replacing entire genes with normal genes, repairing a mutated gene fragment, or making abnormal cells more recognizable by the immune system so they can be effectively removed from the body. Problematic is the delivery of genes using a carrier, usually a viral vector, because viruses can recognize specific cells and introduce genetic material into the cell. Another vehicle may include using of stem cells or liposomes. The risk of gene therapy may be associated with the occurrence of an inappropriate reaction of the immune system, the impact on non-target cells, the appearance of infection with the virus used as a carrier, and even the development of cancer. Liposomal carriers are now very often analyzed. Due to COVID-19, many clinical trials are delayed, but new challenging options are concentrated on producing vaccines and finding new drugs against SARS-CoV-2 using an available portfolio of viral vectors. Usually, adeno-associated virus (AAV) may be chosen to deliver spike protein fragments. Using nanolipids as delivery vectors is also auspicious, not only to transport vaccines but also to deliver specific drugs.

WHO reports 42 candidate vaccines under clinical evaluation: 13 working with protein subunit, 10 using Non-Replicating Viral Vectors, 7 Inactivated, 6 RNA, 4 DNA, and two virus-like particles (VLP). Other 151 candidate vaccines are in preclinical evaluation and use mainly protein subunits (54), Non-Replicating Viral Vectors (18), RNA (18), Replicating Viral Vectors (18), virus-like particles VLP (14), DNA (13), Inactivated (11), Live weakened viruses (3), Replication-competent bacterial vector (1) and based on the use of T cells (1). Only 10 developers/manufacturers of the COVID-19 vaccine have passed phase 3 clinical trials. Among them, the following centers can be distinguished: ii) Sinovac, ii) Wuhan Institute of Biological Products in cooperation with Sinopharm, iii) Beijing Institute of Biological Products in cooperation with Sinopharm as well as iv) CanSino Biological Inc. together with the Beijing Institute of Biotechnology, the following centers can be distinguished: v) the University of Oxford in cooperation with AstraZeneca, vi) Gamaleya Research Institute, vii) Janssen Pharmaceutical Companies, viii) Novavax, ix) Moderna with the National Institute of Allergy and Infectious Diseases (NIAID), and v) BioNTech in cooperation with the Chinese company Fosun Pharma and Pfizer.

The SARS-CoV-2 coronavirus can infect cells with angiotensin-converting enzyme 2 (ACE2) receptors on their surface. It also uses type II transmembrane serine protease TMPRSS2, penetrating, among others, lung epithelial and lung endothelial cells, macrophages, or monocytes. Additionally, coronavirus may use antibody-dependent enhancement (ADE) for infection of cells with a lower level of ACE2 and TMPRSS2 receptors (91). Such a possibility may be connected with problems finding a good strategy for producing vaccines against coronavirus (92). The main aim should be focused on the preparation of specific therapies against SARS-CoV2, including RNA interference (RNAi) (93, 94), small interfering RNA (siRNAs) (95), RNA aptamers, Ribozymes, antisense RNA (ASOs), and oligonucleotide therapeutics (96–99). The ideal vaccine has to be immunogenic with minimal side effects. Production of the vaccine should be efficient and affordable, with the possibility of easy scale-up in full compliance with the principles of good manufacturing practice (GMP). Moreover, the vaccine must not lead to adverse post-vaccination reactions, including antibody-dependent aggravation of infection (100). Conventional methods of obtaining vaccines allow for their effective production. Inactivated vaccines can be used based on attenuated viruses and those using immunogenic subunits. Still, they are associated with the possibility of problems involving strain specificity, risks of viral interference, cross-immunity, allergenicity, or triggering only partially of the immune response. Using genetic vaccines (naked DNA or RNA) like replication-defective recombinant adenoviruses may overcome limitations, be more safely, cost-effective, and quicker, and induce an innate and adaptive immune response, including activation of T cells and antibodies. There is also no problem with the derivation of nucleic acids-based vaccine using antigens sequence and choose fragment, generating better immunological response (88, 100, 101). Genetic vaccines based on DNA and RNA still have some limitations, such as lower immunogenicity. Still, high reproducibility, low costs, and relatively short production time are promising (101, 102).

The nucleic acid-based molecules/drugs may influence viral infection by regulating transcription or post-transcriptional processes, leading to the overexpression of protective genes and silencing damaged genes (103, 104). The main types of nucleic acid-based vaccines are DNA and RNA vaccines outlining in detail the mode of action, evidence supporting a therapeutic strategy based on nucleic acids, the use of new research and development solutions, emerging patents, vaccines for SARS-CoV-2 based on the use of DNA and RNA; clinical trials, expenditures related to the development and production of the vaccine and the pros and cons of vaccines based on mRNA and DNA against SARS-CoV-2were presented in the paper prepared by Piyush et al. (2020) (103).

## 5 Chemical structure, properties, and medical application of tocilizumab

**Figure 3:**
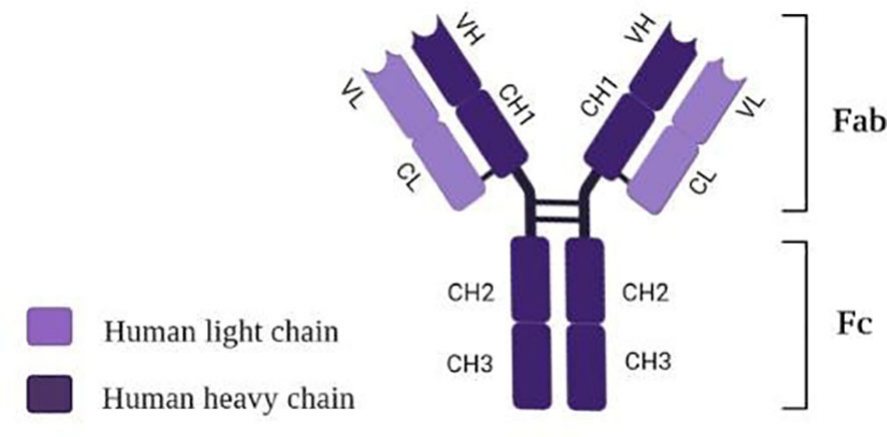
Structure of tocilizumab [own drawing].

Tocilizumab (TCZ), also known as atlizumab (RoActemra^®^), is known as a humanized IgG_1_ monoclonal antibody targeting interleukin-6 (IL-6) receptor (105). The structure of TCZ is schematically shown in **Figure 3**. The antibody consists of two heavy chains (dark violet) containing a variable VH domain and constant domains CH1, CH2, and CH3. In comparison, two light chains (light violet) consist of a variable VL domain and constant CL. Moreover, an antigen-binding fragment (Fab) and a fragment responsible for antibody effectors’ functions (Fc) are distinguished in the structure of this immunosuppressive drug (106).

**Figure 4:**
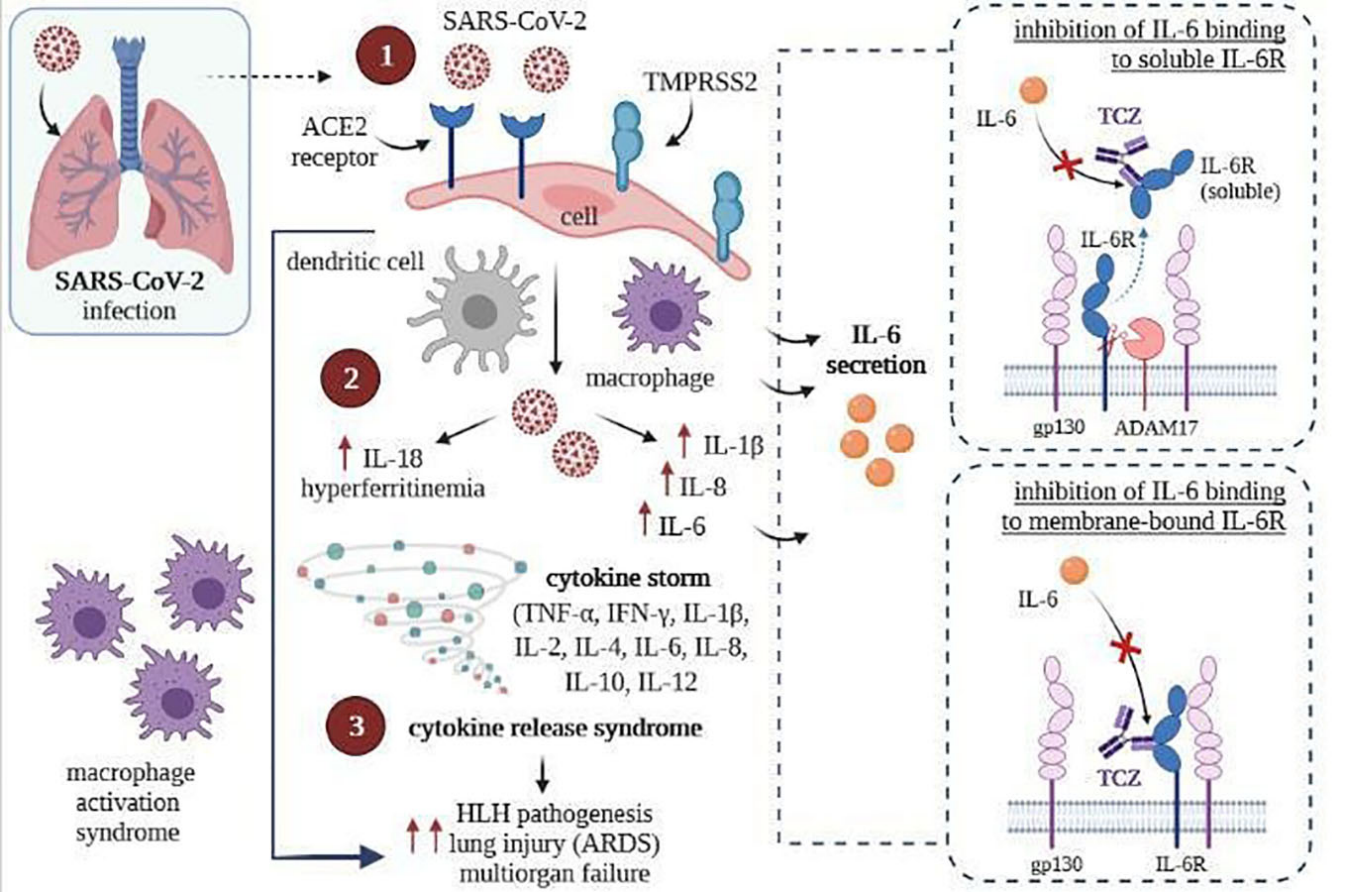
SARS-CoV-2 infection (left) and inhibition of intracellular signaling in cells by TCZ, resulting express of gp130 (right) [own drawing]. (1) Virus entry and infection of pneumocytes expressing the ACE2 receptor, recruiting antigen-presenting cells (dendritic cells and macrophages) into the lungs; (2) Activation of the NLRC4 inflammasome, which causes the overproduction of IL1β and IL 18, and causes the secretion of IL6 and ferritin by macrophages; (3) Upregulation resulting in cytokine release syndrome and macrophage recruitment to the lungs, contributing to ARDS.

Tocilizumab binds with high affinity with both soluble receptors for IL-6 (SIL-6R) and membrane-bound IL-6 receptor (mIL-6R), as well as it inhibits JAK-STAT or MAPK/NF-κB-IL-6 signaling pathway (107, 108). In addition, TCZ can block cytokine storm syndrome (63) and inhibit intracellular signaling in cells expressing soluble gp130 protein (sgp130, **Figure 4**) (109). Currently, this FDA-approved drug commonly used so far in treating rheumatoid arthritis and juvenile idiopathic arthritis is administered intravenously (107, 110) only in hospital conditions, which is a great difficulty in its use during a pandemic.

### 5.1 Tocilizumab in the treatment of cytokine-associated toxicity in COVID-19 patients

Several attempts have been made to use tocilizumab in the active hyperinflammatory phase of COVID-19 (63, 111, 112). Exaggerated immune response to infection with the SARS-CoV-2 virus contributes to respiratory distress and multi-organ failure (113). It is caused by the elicitation of the so-called cytokine storm (114) (**Figure 4**). The results of recent scientific reports have shown that TCZ therapy in COVID-19 can drive a significant reduction in the inflammatory process. It can be explained by the induction of apoptosis of immunocompetent cells in affected tissues and by inhibiting proinflammatory cytokine release (115–117). Although the results are promising, previous studies of cytokine storm associated with other coronavirus and influenza virus infections and CAR (chimeric antigen receptor)-T cell therapy have also proven high levels of interleukin (IL)-6 and other cytokines (113, 114).

The study by Guo et al. (2020) has shown that immunosuppressive drugs targeting the IL-6 receptor, such as TCZ, can ameliorate lethal inflammatory responses in COVID-19 patients (118). The research has proven that the inflammatory cascade caused by excessive immune responses correlated with the death rate of COVID-19 (83, 119). As a result of SARS-CoV-2 infection, an increase in plasma concentrations of several inflammatory cytokines was observed, besides tumor necrosis factor α (TNF-α), interleukins (IL-2,-6,-7,-10), granulocyte-macrophage colony-stimulating factor (GM-CSF), and granulocyte colony-stimulating factor (G-CSF) (118).

Since tocilizumab has been considered effective for treating severe cytokine-release syndrome (120), this immunosuppressive drug has also been applied to treat selected COVID-19 patients (121). Other results have shown that pathogenic T cells and peripheral inflammatory monocytes may induce cytokine-associated toxicity in patients infected with SARS-CoV-2. However, the administration of tocilizumab decreased the patient’s body temperature within 24  hours, and a visible reduction of oxygen inhalation in COVID-19 patients within less than a week of treatment (122). Although it was shown that TCZ might efficiently attenuate the cytokine cascade in COVID-19 patients, no single-cell-level analysis explaining these phenomena has been performed so far. This study would help to disclose the tocilizumab mode of action in the context of a characteristic COVID-19-induced activation of an inflammatory storm (118).

Xu et al. (2020) conducted an uncontrolled study using TCZ as IL- 6 blocker in 21 COVID-19 patients with the most common symptoms. All patients required supplemental oxygen (2 were on ventilators), had worsening ground-glass opacities on chest computed tomography, and showed deterioration of other clinical and laboratory measures (122). It is worth underlining that within 24 hours of TCZ therapy beginning, fevers and increased C-reactive protein levels significantly resolved, while all pro-inflammatory cytokines (especially IL-6) declined. Furthermore, there was no urgent need to use oxygen in 15 patients. In all patients, oxygen saturation levels were improved (122).

One of the most extensive studies regarding the use of tocilizumab in COVID-19 patients was conducted in Northern Italy and reported by Guaraldi et al. (2020) (105). SARS-CoV-2-infected patients were administered 8 mg/kg (up to 800 mg) of TCZ intravenously or 162 mg subcutaneously in two simultaneous doses (81 mg per thigh). Both amounts were based on pharmacokinetic data and were intended to mimic peak plasma concentration (111). Patients who received tocilizumab were compared with a control group with the same inclusion and exclusion criteria. However, the main limitation of this study was that patients and controls were not randomly chosen, thus making it impossible to compare the obtained results and draw reliable conclusions.

Another study has identified the outcomes among SARS-CoV-2-infected patients treated with tocilizumab to target cytokine storms (112). The results helped set specific criteria to define the cytokine storm in all SARS-CoV-2-RNA-positive patients. Moreover, it has been shown that early identification and inhibition of cytokine storms before intubation is much more significant than any anti-inflammatory treatment. Cytokine storm duration should be included, while randomized controlled trials based on targeted anti-cytokine and corticosteroids may also be considered (109, 112, 123).

## 6 The impact of the endocannabinoid system on COVID-19

Emerging reports on the production of endocannabinoids in the respiratory system and cannabinoid-induced bronchial dilatation allow conclusions about the potential therapeutic use of cannabinoids in treating respiratory diseases, including acute respiratory failure syndrome in severe COVID-19 patients (124). Despite the identification of the first strains of human coronavirus in the 1960s and the molecular similarity of SARS-CoV to SARS-CoV-2, no studies have been conducted to prove the effect of cannabinoids on this family of single-stranded RNA viruses so far. There was also no objective evidence of the therapeutic, anti-inflammatory effects of cannabidiol (CBD) and delta-9-tetrahydrocannabinol (Δ^9^-THC), the two main cannabinoids would contribute to promote or prevent the application of cannabinoids as compounds to the fight against the virus. Nonetheless, it seems that THC, CBD, or other cannabinoids can act as immune modulators, which could be helpful in the treatment of viral infections, especially those where we have a pathogenic host-inflammatory response, as with SARS-CoV-2 (125).

Infection caused by SARS-CoV-2 leads, for reasons not fully explained, to the overproduction of inflammatory cytokines (mainly from immune cells) with a wide range of biological activity, which is caused by various infections and loss of unfavorable influence on the immune system. On the other hand, these cytokines positively influence different immune cells, guiding them to the inflammation sites, which causes an exponential increase in inflammation, leading to continuous extreme activation of the autoimmune system. This mechanism is called a cytokine storm, a significant cause of acute respiratory distress syndrome, systemic inflammatory response, and multi-organ failure (126). It is suggested that cannabinoids could be part of two schemes to treat these acute inflammatory reactions. The first, with non-steroidal anti-inflammatory drugs (NSAIDs) targeting the immune system, which hurt antiviral therapies due to NSAIDs interactions and weakening the immune response to acute viral infections leading to disease progression, while the second, with drugs specifically binding to pro-inflammatory cytokine receptors, such as tocilizumab that inhibits the transmission of the signals through IL-6 receptors leading to a weakening of IL-6 activity.

## 7 Cannabinoids and their influence on the treatment of COVID-19

Cannabidiol (CBD; C_21_H_30_O_2_) is a phytocannabinoid without psychoactive activities produced by *Cannabis Sativa* L and has a structural similarity to Δ^9^- tetrahydrocannabinol (THC; C_21_H_30_O_2_), the primary psychotropic congener of this cannabis plant. Both cannabinoids are lipophilic compounds characterized by long half-life, bioaccumulation, and shared common metabolic pathways within the cytochrome family, drug carriers, and plasma protein binding substrates (127). Both of them also have anti-inflammatory activity and possible antiviral potential. Nonetheless, the majority of studies indicate the immunosuppressive and anti-inflammatory effects of cannabidiol in various immunological reactions and inflammations (70). CBD, unlike THC, is a non-toxic compound with a high safety margin and drug tolerance, even at doses up to 1500 mg/day. Recently, it was demonstrated that cannabidiol has anti-inflammatory effects in chronic inflammatory diseases preclinical models, apoptotic effects on the mammalian cells (70), or effects that contribute to the host of the viral infection response (128, 129).

Interleukin-6 is effectively suppressed by cannabidiol in numerous models of inflammation, including diabetes, asthma, pancreatitis, and hepatitis. *In vivo*, cannabidiol use resulted in an IL-6 decrease in ex vivo lipopolysaccharide-stimulated peritoneal macrophages in acute pancreatitis and bronchial-alveolar lavage fluid in lipopolysaccharide-induced pneumonia. Also, in mice with the induced asthma-like disease, IL-4, IL-5, and IL-13 cytokines and chemokines caused in the lungs of mice were shown to be suppressed by CBD (70). Cannabidiol decreased lung inflammation in asthma and acute pneumonia mouse models by inhibiting the production of pro-inflammatory cytokines by immune cells and suppressing the exuberant immune response (130, 131). Nichols and Kaplan (2020) (70) have shown that CBD inhibits the production of pro-inflammatory cytokines such as interleukin IL-1α and β, IL -2, IL-6, Il-17A interferon-γ inducible protein 10, monocyte chemoattractant protein-1, tumor necrosis factor -α and macrophage inflammatory protein-1α, which are associated with the occurrence of polyorgan inflammation and high mortality caused by SARS-CoV-2 (70). The current research results point out that CBD’s immunotherapeutic and anti-inflammatory properties may limit the cytokine storm and reduce the effects of exaggerated inflammation in patients with severe respiratory tract viral infections and ARDS often associated with COVID-19 (124).

**Figure 5:**
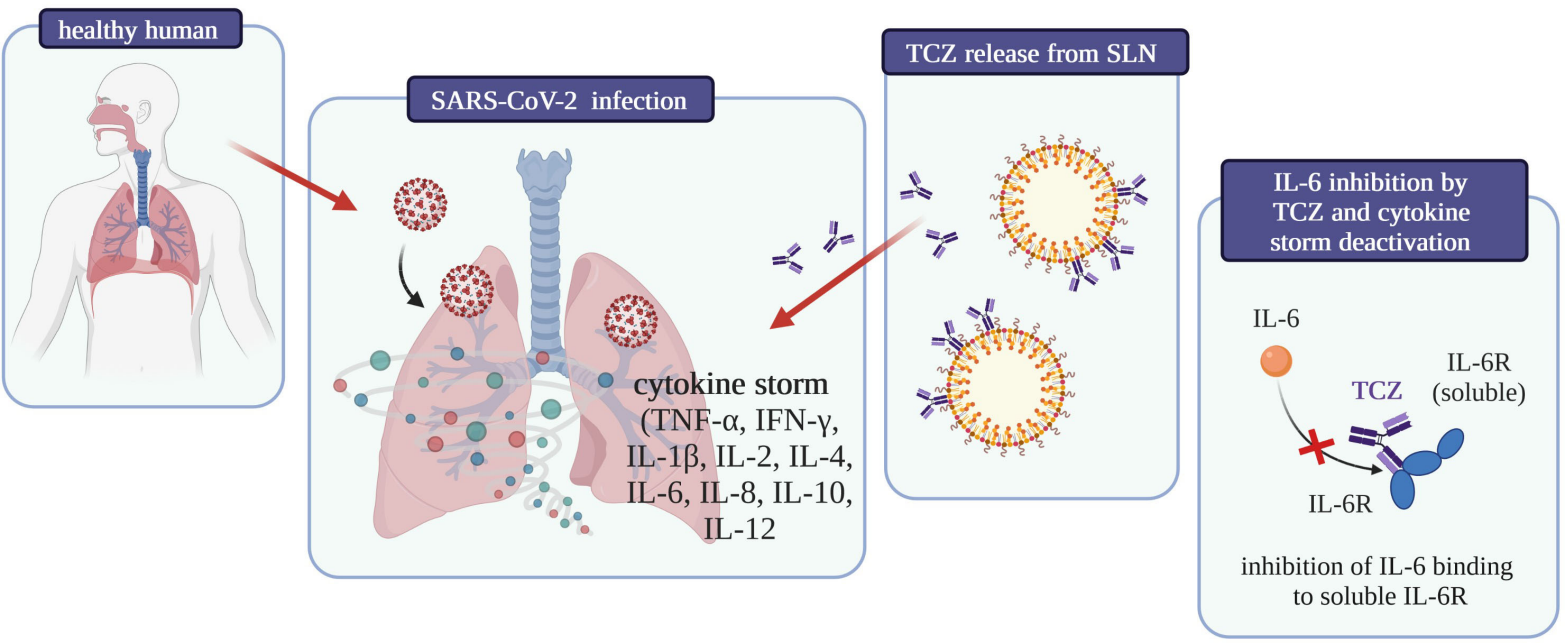
Graphical summary of the influence on COVID-19 treatment of tocilizumab-coated solid lipid nanoparticles [own drawing].

To date, no studies about interactions between drugs used to treat SARS-CoV-2 infection and CBD have been published, but considering the inhibition of interleukin 6 receptor by Tocilizumab and the anti-inflammatory role of cannabidiol in the treatment of severe respiratory viral infections, a synergic, significant effect of these compounds on the reduction of inflammation in the acute course of COVID-19 can be expected (**Figure 5**).

The constant mutations of SARS-CoV-2 make it imperative to identify an effective medication for patients suffering from COVID -19. The possibility of using cannabinoids to treat severe cases of this disease is increasingly being discussed.

The novel coronavirus binds to cellular receptors *via* angiotensin-converting enzyme 2 (ACE2), characteristic of pulmonary tissue, oral and nasal mucosa, kidneys, IG, and testicles. Furthermore, smokers and patients with chronic obstructive pulmonary disease have been reported to be more susceptible to COVID-19 and develop a severe form of the illness, as they present a high level of ACE2 expression. Therefore, it is believed that ACE2 expression in the oral, respiratory, and intestinal epithelium may provide SARS-CoV-2 with a vital entry point into the host cells, and ACE2 modulation in these tissues may limit SARS-CoV-2 binding to ACE2 receptors and thus reduce susceptibility to COVID-19 (57, 132–134). A study by Wang et al. on artificial human 3D models of the tissues mentioned above allowed these authors to identify 13 extracts from *Cannabis sativa* with a significant amount of CBD, which affects the expression of the ACE2 gene and the level of the ACE2 protein. Preliminary experiments also demonstrated that the extracts studied may decrease the concentration of the transmembrane protease serine 2 (TMPRSS2), a protein essential in the process of the viruses entering into cells (57).

The cannabinoid system consists of two cannabinoid receptors: CB1, present in the central nervous system (CNS), and CB2, in the immune system. Various ligands activate cannabinoid receptors: endogenous (anandamide, AEA; 2-arachidonoylglycerol, 2-AG), exogenous (e.g., phytocannabinoids from *Cannabis sativa* L.), or synthetic (135). The most critical phytocannabinoids include psychoactive delta-9-tetrahydrocannabinol (THC) and non-psychoactive cannabidiol (CBD). In contrast to THC, a partial antagonist of both CB1 and CB2 receptors, CBD is a partial antagonist of CB2 and only a weak antagonist of CB1 (136–138). Other cannabinoids are found in dried cannabis in much lower amounts. Cannabigerol and cannabichromene inhibit AEA re-uptake (139). The MOA of numerous other phytocannabinoids, such as cannabidivarin, cannabidiol, or cannabielsoin, has not been fully explored yet (138). When developing an adequate preparation based on cannabis flower extracts, it must be remembered that cannabis contains over 100 identified cannabinoids, Δ^9^-THC and CBD being the best-known ones, and other compounds like terpenes (140, 141). Terpenes and cannabinoids may interact with each other, affecting a given extract’s overall therapeutic effect. It is assumed that a quote is more potent than a single compound; therefore, it is essential to study the impact of a whole extract obtained from a plant rather than a single compound (142, 143). It has been known that anandamide (AEA) endocannabinoid, an endogenous antagonist with a high affinity to CB1, decreases IL-6 production. In contrast, THC, a partial antagonist of CB1 and CB2, inhibits IL-12 and IFN-γ release (144–148). Another phytocannabinoid (E)-β-caryophyllene [(E)-BCP], as a functional antagonist of CB2, inhibits both the production of pro-inflammatory cytokines in the peripheral blood induced by lipopolysaccharides (LPS) and the LPS-incited phosphorylation of Erk1/2 and JNK1/2 in monocytes (149–151). CBD impedes the expression of Il-6, IL-8, and TNF-α in *in vitro* models of allergic contact dermatitis and bone and joint inflammation. On the other hand, delta-9-tetrahydrocannabinol reduces the release of TNF-α, IL-1β, IL-6, and IL-8 in MG63 cells incited with LPS, which points to an essential role of the CB2 receptor in the anti-inflammatory response (152, 153). COVID-19 patients show macrophages, monocytes, and low levels of lymphocytes accountable for acute lung injury and leading to acute respiratory distress syndrome or even death. There are two groups of macrophages involved in the inflammatory response, cytokine production, phagocytosis, cell proliferation, and tissue repair: classically activated macrophages (M1) and alternatively activated macrophages (M2) (154–157). CB2 receptors are known as macrophage polarization regulators in inflammation. The use of an antagonist reduces the proliferation of inflammation-stimulating macrophages (M1) and increases the commonness of the second type of macrophages, which have an opposite effect (M2) (148, 158).

In SARS-CoV2 infection, there is a change in cytokine production, very similar to a cytokine storm, accompanied by excessive release of immune cells. Mesenchymal stromal cells (MSCs) have an anti-inflammatory effect. Their use may decrease the production of inflammatory-inducing compounds, which could improve the condition of the lungs previously damaged by, e.g., the flu virus (64, 159–161). MSCs raise the level of peripheral lymphocytes, simultaneously lowering the number of immune cells producing cytokines. Furthermore, MSCs produce leukemia inhibitory factor (LIF), which helps counteract the cytokine storm in viral pneumonia and stimulates CB2 receptors (162–164). The proposed MSC treatment for COVID-19 patients and proper stimulation of CB2 receptors will allow for the repair of damaged stem cells and immune response stimulation. It has been observed that MSCs do not stimulate the synthesis of ACE2 and TMPRSS2 proteins, which are associated with SARS-CoV-2 infection (165–167).

It was demonstrated that, in comparison to women, men are more prone to SARS-CoV-2 infection, which lower estrogen concentrations could explain. This beneficial property of estrogens and the immunosuppressive effect, which reduces excessive inflammation, may be associated with the CB2 cannabinoid receptor, a well-known immune response modulator (168–172).

Selective stimulation of CB2 may limit inflammation in COVID-19 patients through inflammatory cascade control in several checkpoints by reducing cytokine production, limiting immune cell proliferation, or producing antibodies, thus eliminating acute immune response (148, 173, 174). Currently, there are no CB2 antagonists approved for human use. Therefore, while searching for a commercially available CB2 receptor inhibitor is ongoing, treatment with CBD may be an alternative solution for COVID-19 patients (148).

Numerous experimental studies on rodents have shown that CB1 activation is essential for an effective immune response in bacterial infections, whereas CB2 activation prevents further damage caused by inflammation in sepsis due to an immunosuppressive effect (175). El Biali et al. (2020) (175) report a few human studies identifying potential relationships between the endocannabinoid system and the immune response. For instance, it was found that a genetic polymorphism in CB2 (CBQ63R), which reduces CB2 responses, can be linked with a higher probability of hospitalization in small children infected with RSV (n = 83), with the risk of developing severe ARTI being two times higher in allele Q carriers (OR = 2.148; 95% CI:1.09–4.22), and three times higher in the carriers of the QQ genotype (OR = 3.28; 95% CI: 1.22–8.71) (175, 176).

The FDA approved two forms of delta-9-tetrahydrocannabinol (dronabinol and nabilone) for treating some of the adverse effects of chemotherapy (i.e., nausea and vomiting) and for recovery of the appetite in wasting diseases like AIDS. In 2018, CBD was permitted for treating two types of pediatric epilepsy: Dravet syndrome and Lennox-Gastaut syndrome. Apart from these four indications, the most solid evidence of using cannabinoids with desired therapeutic outcomes is observed in chronic pain (including neuropathic pain) and MS-related muscle spasticity (125). Numerous *in vitro* and *in vivo* studies on animal and human cells suggest that CBD has an immunosuppressive and anti-inflammatory effect through direct inhibition of microglial cells and T cells, induction of apoptosis in regulatory T cells, or through myeloid-derived regulatory T cell induction (70). *In vitro* studies in animals suggest that *Cannabis sativa* extracts have an anti-bacterial and limited anti-fungal properties (177). A study conducted among healthy volunteers (n = 10) demonstrated that a dose of 30 mg of water-soluble or fat-soluble CBD significantly decreased the TNF level in peripheral blood mononuclear cells stimulated with bacterial lipopolysaccharide (178).

To date, there have been no studies investigating the effect of cannabinoids on SARS-CoV-2 infection. Furthermore, there is no epidemiological data on COVID-19 incidence in people using cannabinoids for medical or recreational purposes. A paper by Esposito lists four features of CBD that warrant its use, namely: 1) *Cannabis sativa* extracts have been proven to regulate the expression of two receptors that are of crucial importance for SARS-CoV-2 in a cellular model, 2) it has been demonstrated that CBD has an extensive range of immunomodulatory and anti-inflammatory effects, which may reduce the over-production of that leads to acute lung injury, 3) as a PPARγ antagonist, CBD may present a direct anti-viral effect, 4) as a PPARγ antagonist, CBD may inhibit the process of pulmonary fibrosis (179).

The characteristic feature of severe SARS-CoV-2 infection is the uncontrolled release of cytokines IL-1β, IL-6, and CCL2, and pro-inflammatory molecules, together with a decrease in the number of NK (natural killer) cells that may cause the cytokine storm. There is much to suggest that the severe course of infection does not stem from the viremia *per se* but depends on the degree of immune dysregulation. To limit mortality in severe cases of COVID-19, it is necessary to develop new therapeutic options to mitigate the cytokine storm (180). Esposito et al. (2020) indicate that due to the rapid spread of the pandemic, the ideal drug candidate should already be used in treating other diseases, have a good safety profile, and act to mitigate the cytokine storm through immunomodulation rather than immunosuppression (179). Recently, it was shown that *Cannabis sativa* extracts with a high concentration of CBD down-regulate the activity of ACE2 and TMPRSS2 enzymes, which are vital for SARS-CoV-2 to enter the human body (181). In recent years, CBD has been the subject of much research due to its broad spectrum of therapeutic effects, including anti-seizure, calming, sleep-inducing, anti-psychotic, anti-cancer, anti-inflammatory, and neuroprotective effects (182). What has been emphasized is the lack of adverse psychotropic effects of cannabidiol and its favorable safety profile in humans (182). The pharmacological activity of CBD was tested in patients suffering from various conditions, including respiratory diseases characterized by acute lung injury (179). CBD effect on adenosine A2A receptors limited leukocyte migration to the lungs, which was accompanied by a significant decrease in pro-inflammatory cytokine (TNF-α and IL-6) and chemokine (MCP-1/MIP-2/CXCL2) release, thus clearly improving the compromised lung function (131, 183).

CBD has also been studied as a molecule with a potential anti-viral effect. CBD acts by interacting with nuclear hormone receptors PPAR. It was shown that down-regulation of PPARγ expression by alveolar macrophages significantly reduces lung inflammation and enhances regeneration after viral respiratory tract infections (184). Preventive and therapeutic administration of PPARγ antagonists decreased morbidity and mortality related to influenza A virus infection (185). However, the use of full PPARγ agonists has several adverse effects, including the risk of cardiovascular complications, cardiac insufficiency, and stroke. It needs to be verified whether CBD, as a weak antagonist of PPARγ, could be used without causing such adverse effects (179). There is no direct proof of the anti-viral activity of cannabinoids in viral infections. Such products have been described *in vitro* studies. Lowe et al. demonstrated an anti-viral effect of CBD against the hepatitis C virus (HCV), but not against HBV, in cell lines for producing these viruses (128). Anti-viral activity of CBD was also confirmed against Kaposi’s sarcoma-associated herpesvirus (KSHV) in a model of KSHV-infected human dermal microvascular endothelial cells (HMVECs) (129). In yet another study, CBD mitigated the effects of neuroinflammation induced by Theiler’s murine encephalomyelitis virus (TMEV) (186). Respiratory syncytial virus (RSV) used in the mouse model has indicated that CB2 activation reduced infection symptoms, and CB1 antagonist administration alleviated pulmonary complications (176).

Research has shown that CBD is a reasonably safe molecule (187). In the case of COVID-19 patients, it is essential to establish the toxicity profile of CBD when administered concomitantly with other drugs used in the current anti-COVID-19 protocols.

First, pharmacokinetic (PK) and pharmacodynamic (PD) interactions between cannabinoids and experimental COVID-19 drugs must be determined. Both Δ^9^-THC and CBD are lipophilic, highly protein-bound molecules. They have a long half-life, undergo bioaccumulation, and share metabolic pathways with cytochrome P450, drug transporters (e.g., breast cancer resistance protein), and substrates that bind to plasma proteins (127). Furthermore, PK (e.g., warfarin and clobazam) and PD (e.g., valproic acid) interactions were described for THC and CBD (188). Land et al. (2020) (127) compiled a table with 16 compounds examined for their potential use in COVID-19 treatment and their possible pharmacokinetic or pharmacodynamic interactions with cannabinoids.

They found that most candidate drugs could interact with THC and CBD. The authors propose that COVID-19 patients should be asked about their use of substances containing cannabinoids, as these may significantly affect their reaction to the selected treatment method (127).

Esposito points to the possibility of testing the therapeutic potential of CBD in COVID-19 patients at the beginning of the disease to suppress the cytokine storm, prevent the danger of respiratory failure, or assess the effect of CBD on pulmonary fibrosis. The central aspect of being clarified is dosing. In the case of HIV and post-Ebola syndrome, CBD was used as an agent controlling immune activation at doses of 10–20 mg · kg^−1^ · day^−1^ and 1.7–10 mg · kg^−1^ · day^−1^ (100 mg · day^−1^ titrating up to 600 mg · day^−1^) (189, 190).

The results of pre-clinical studies are encouraging. However, evidence is needed to approve cannabidiol as a supportive drug in COVID-19 treatment.

## 8 Lipid nanoparticles as carriers for proteins and monoclonal antibodies

Lipid nanoparticles (LNPs) are composed of biodegradable and biocompatible lipids (191, 192), and they can be successfully proposed to encapsulate proteins (193–198). The literature describes two classical types of lipid nanoparticles, namely:

- **solid lipid nanoparticles (SLN)**, so-called “1st generation”,
- **nanostructured lipid carriers (NLC)**, so-called “2nd generation”.

SLN and NLC are known to increase the bioavailability of loaded drugs administered by different routes (199, 200). In contrast to SLN, the lipid matrix of NLC consists of a mixture of solid and liquid lipids (that melt above 40°C). The matrix originates a less-ordered matrix with the capacity to load a higher drug amount than SLN, preventing its leakage during storage and allowing a more flexible drug release modulation (201). SLN contains only solid lipids in its matrix, offering the capacity to modulate the release profile of loaded drugs. SLN and NLC require surfactants (e.g., poloxamers, tweens) to stabilize the lipid matrices in aqueous dispersion (202, 203). Acylglycerols, waxes, fatty acids, and hard fats are the most commonly used lipids that should be approved by the Food and Drug Administration (191, 204).

### 8.1 Drug release form of SLN

There are three basic types of SLN, defined by the location of the drug in the lipid matrix (205, 206). Loaded drugs can be placed between fatty acid chains or between lipid layers. The final drug location affects its release mechanism from the lipid matrices (198).

The SLN type I is defined as the homogeneous matrix model, in which the drug is molecularly dispersed in the lipid core or amorphous clusters. This model is obtained when applying the hot, high-pressure homogenization (HPH) in an optimized drug and lipid ratio or when using the cold HPH. Due to their structure, SLN type I can show controlled release properties.

The SLN type II, or drug-enriched shell model, is obtained when applying the hot HPH technique and the low drug concentration in the melted lipid. During the cooling of the homogenized nanoemulsion, the lipid molecules precipitate first. Then, they lead to a steadily increasing drug concentration in the remaining lipid melt with an increased fraction of solidified lipid. A drug-free (or drug-reduced) lipid core is formed; when the drug reaches its saturation solubility in the remaining melt, an outer shell will solidify, containing both drug and lipid. This model is not suitable for prolonged drug release.

**Figure 6:**
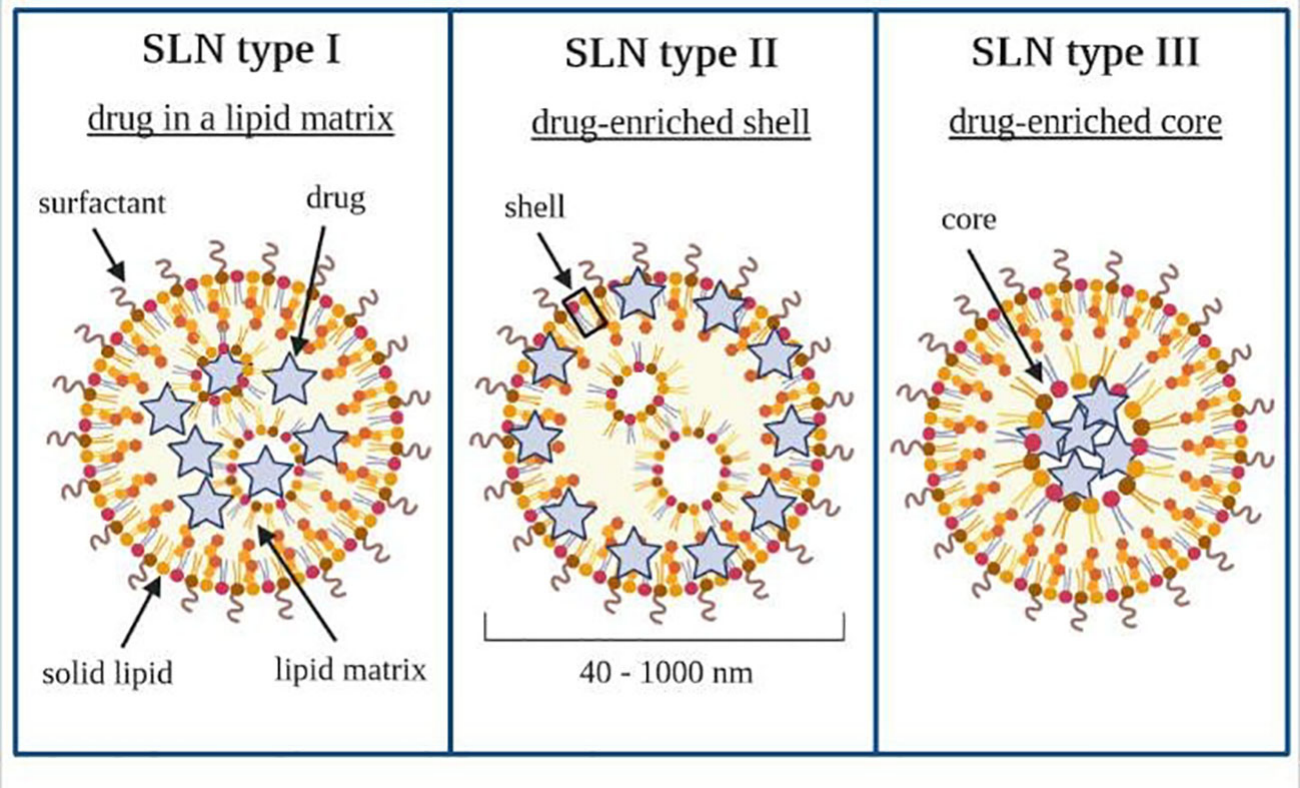
Types of solid lipid nanoparticles. Own drawing based on (206).

Nonetheless, it may be used to obtain a burst release of medicine and the occlusive properties of the lipid core. The SLN type III, or drug-enriched core model, is formed when the drug concentration is relatively close to or at its saturation solubility in the lipid melt. Under the nanoemulsion cooling, the drug’s solubility will decrease when the saturation solubility is exceeded. This model is also helpful for prolonged-release purposes (204, 207–209). SLN work as an absorption enhancer when orally administered (206, 210, 211). Types of SLN that can be obtained have been shown schematically in **Figure 6**.

### 8.2 TCZ-coated SLN loaded with CBD

The use of CBD-based SLN as potential TCZ carriers for oral administration is mainly associated with the safety of LNPs and their ability to faster enteral administration with the increased bioavailability of both hydrophilic and lipophilic active substances. On the other hand, understanding the impact of the size and shape of LNPs on their distribution in the intestine can be used in developing improved drug delivery systems to treat COVID-19. The biodegradable lipid matrix of SLN undergoes enzymatic decomposition into components naturally occurring in the human body (191). The novel approach proposed by the authors is the oral form administration of TCZ and CBD simultaneously, which can significantly improve the comfort of patients who have previously used regular intravenous injections of mAb. Moreover, the obtained SLN may be an ideal carrier for TCZ/CBD because of the ability of LNPs to modify drug release, increase bioavailability and thus regulate pharmacological activity (212). The selection of the type of lipids to be used for the production of SLN is governed by the solubility of CBD in the solid lipid. We have recently developed a stabilized SLN formulation based on glycerol behenate for the loading of CBD. The surface tailoring with the mAb is usually carried out by biotinylating as described by Souto et al. (2019) (213). Due to the potential of SLN to delay drug release, there is a high probability of optimizing these nanocarriers for drug release in the colon, thereby protecting the gastrointestinal tract against the destructive influence of COVID-19 at the initial phase. It is worth underlining that a final enteric formulation would be developed for the delayed release of the actives into the colon by encapsulating drugs-loaded LNPs in gastro-resistant capsules to prevent earlier degradation of nanoparticles in the stomach (214).

The development of surface-modified SLN formulations for targeted delivery to the colon requires the production of gastro-resistant capsules in which the TCZ-coated CBD-loaded SLN dispersions are loaded. Enteric coatings for colonic administration exploit the pH differences along the gastrointestinal tract to release the drug only when reaching pH 6.0-7.0 in the colon. Polymethacrylates (e.g., Eudragit^®^ brands) are typical polymers that coat tablets and capsules to protect the drugs from gastric and small intestinal pathways. They are commonly found in commercially available pharmaceutical formulations for ulcerative colitis and Crohn’s disease (215).

The combination of SLN and electroporation has been proposed to enhance drug transport to the colon. Cyanine–type IR 780 and the flavonoid derivative baicalein were co-loaded into SLN for both imaging and therapy of colorectal carcinoma (216). The authors reported that the presence of flavonoids contributed to reducing the dose and, thus, cytotoxicity in chemotherapy. Electroporation generates external electric field pulses and increases cell membrane permeability, which offers the opportunity for intracellular trafficking of the cargo (217). The p53 and manganese superoxide dismutase expression was significantly increased by electroporation, with substantially higher cytotoxicity.

## 9 Conclusion

In this review, scientific evidence is given about the added value of using cannabidiol (CBD) for the management SARS-CoV-2 infection. CBD was found to down-regulate the activity of ACE2 and TMPRSS2 enzymes, both governing the entry of SARS-CoV-2 in the human body. Besides reducing the secretion of cytokines, cannabidiol also promotes body protection against several pathogenic infections and is also expected to can play a similar role against COVID-19. On the other hand, solid lipid nanoparticles (SLN) are ideal carriers for the oral administration of drugs, provided that lipids are known to be absorption enhancers in the gastrointestinal tract. Given its lipophilic character, CBD is an appropriate candidate to be loaded into SLN. A synergistic effect is expected with the combination of tocilizumab (TCZ), as this monoclonal antibody is an anti-IL-6 receptor antibody in COVID-19 treatment. Indeed, cytokine-associated toxicity is linked to pro-inflammatory cytokine IL-6 released in severe COVID-19 infections. An innovative approach is thus proposed by exploiting the advantages of coating SLN with mAb for site specific targeting. To obtain a suitable pharmaceutical dosage form for the oral administration of TCZ-coated CBD-loaded SLN, further studies are suggested towards the optimization of gastro-resistant gelatin capsules in which the lipid nanoparticle formulations can be encapsulated.

## Author contributions

AZ, PE, JK, and EBS were responsible for conceptualizing the manuscript. AZ, PE, JK, MaS, SH, KW, MiS, and EBS were responsible for writing and editing the paper. AD, AGA, RS, and ES were responsible for reviewing, while AZ was responsible for the visualization of the manuscript. All authors have made substantial contributions to the conception and design of the paper, drafting the article and revising it critically for important intellectual content, and final approval of the version to be submitted. All authors contributed to the article and approved the submitted version.
