# Supplementary material for: Tocilizumab-coated solid lipid nanoparticles loaded with cannabidiol as a novel drug delivery strategy for treating COVID-19: A review
Source: Front Immunol. 2023 Mar 22;14:1147991. doi: 10.3389/fimmu.2023.1147991 (PMC10073701; doi:10.3389/fimmu.2023.1147991)
Supplement: Supplementary file 1 [file DataSheet_1.pdf]

Table 1. Potential phytochemicals for antiviral drug development.

Phytochemical name	Plant source
5,7,3′,4′-Tetrahydroxy-2'-(3,3-dimethylallyl) isoflavone	Mojave indigo bush *(Psorothamnus arborescens)*
Myricitrin	Wax myrtle (*Myrica cerifera*)
Methyl rosmarinate	Marubio oscuro (*Hyptis atrorubens Poit)*
3,5,7,3′,4′,5′-hexahydroxy flavanone-3-O-beta-D-glucopyranoside	Common bean (*Phaseolus vulgaris*)
(2S)-Eriodictyol 7-O-(6″-O-galloyl)-beta-D-glucopyranoside	Indian gooseberry (*Phyllanthus emblica*)
Calceolarioside B	Chinese flowering ash (*Fraxinus sieboldiana*)
Myricetin 3-O-beta-D-glucopyranoside	Tea tree (*Camellia sinensis*)
Licoleafol	Chinese liquorice (*Glycyrrhiza uralensis*)
Amaranthin	Edible amaranth (*Amaranthus tricolor*)
